# CRFB5a, a Subtype of Japanese Eel (*Anguilla japonica*) Type I IFN Receptor, Regulates Host Antiviral and Antimicrobial Functions through Activation of IRF3/IRF7 and LEAP2

**DOI:** 10.3390/ani13193157

**Published:** 2023-10-09

**Authors:** Tianyu Wang, Peng Lin, Yilei Wang, Xiaojian Lai, Pengyun Chen, Fuyan Li, Jianjun Feng

**Affiliations:** 1 Key Laboratory of Healthy Mariculture for the East China Sea, Ministry of Agriculture and Rural Affairs, Fisheries College, Jimei University, Xiamen 361021, China; 202111908006@jmu.edu.cn (T.W.); linpeng@jmu.edu.cn (P.L.); ylwang@jmu.edu.cn (Y.W.); laixj@jmu.edu.cn (X.L.); 202011908033@jmu.edu.cn (P.C.); 18288798950@163.com (F.L.); 2 The Open Program of Key Laboratory of Cultivation and High-Value Utilization of Marine Organisms in Fujian Province, Xiamen 361000, China

**Keywords:** *Anguilla japonica*, CRFB5a, RNAi, type I IFN, LEAP2

## Abstract

**Simple Summary:**

In this study, we investigated the response mechanism of type I interferon receptor in the Japanese eel (*Anguilla japonica*) after an immune challenge, and we also explored the key role of type I interferon receptor in its immune response mechanism, which will provide new evidence for the study of type I interferon receptor in vertebrates, especially in teleost.

**Abstract:**

IFNAR1, one of the type I IFN receptors, is crucial to mammalian host defense against viral invasion. However, largely unknown is the immunological role of the fish teleost protein IFNAR1, also known as CRFB5. We have successfully cloned the whole cDNA of the Japanese eel’s (*Anguilla japonica*) CRFB5a homolog, *Aj*CRFB5a. The two fibronectin-3 domains and the transmembrane region (238–260 aa) of *Aj*CRFB5a are normally present, and it shares a three-dimensional structure with zebrafish, Asian arowana, and humans. According to expression analyses, *Aj*CRFB5a is highly expressed in all tissues found, particularly the liver and intestine. In vivo, *Aeromonas hydrophila*, LPS, and the viral mimic poly I:C all dramatically increased *Aj*CRFB5a expression in the liver. Japanese eel liver cells were reported to express *Aj*CRFB5a more strongly in vitro after being exposed to *Aeromonas hydrophila* or being stimulated with poly I: C. The membranes of Japanese eel liver cells contained EGFP-*Aj*CRFB5a proteins, some of which were condensed, according to the results of fluorescence microscopy. Luciferase reporter assays showed that *Aj*CRFB5a overexpression strongly increased the expression of immune-related genes in Japanese eel liver cells, such as IFN1, IFN2, IFN3, IFN4, IRF3, IRF5, and IRF7 of the type I IFN signaling pathway, as well as one of the essential antimicrobial peptides LEAP2, in addition to significantly inducing human IFN-promoter activities in HEK293 cells. Additionally, RNA interference (RNAi) data demonstrated that knocking down *Aj*CRFB5a caused all eight of those genes to drastically lower their expression in Japanese eel liver cells, as well as to variable degrees in the kidney, spleen, liver, and intestine. Our findings together showed that *Aj*CRFB5a participates in the host immune response to bacterial infection by inducing antimicrobial peptides mediated by LEAP2 and favorably modulates host antiviral immune responses by activating IRF3 and IRF7-driven type I IFN signaling pathways.

## 1. Introduction

Interferon (IFN) is noted as a crucial cytokine of glycoproteins exhibiting multiple activities in anti-virus, anti-tumor, and immunomodulatory effects [1]. In mammals, based on cell origin, sequence homology, and different receptor system inducible factor types [2], IFNs have been classified into three groups: type I IFN (IFN-α, IFN-β, IFN-δ, IFN-ε, IFN-ζ, IFN-κ, IFN-τ, IFN-ω), type II IFN (IFN-γ), and type III (IFNλ) [3,4,5]. Type I IFN plays a central role in host antiviral immunity, in which IFN-α and IFN-β as two classical Type I IFNs were found to first bind to their receptors and then initiate the JAK/STAT signaling pathway, resulting in the expression of Mx proteins and other antiviral proteins [6]. To date, two subunits of type I interferon receptors, IFNAR1 and IFNAR2, have been identified in humans [7]. Compared with IFNAR1, IFNAR2 has a higher affinity for interferon. IFNAR2 can first combine with interferon and then associate with IFNAR1 to form a complex leading to the activation of the JAK/STAT signal pathway [8,9]. Further studies showed that the change in the expression level of IFNAR2 mainly affects the activation level of the JAK/STAT signaling pathway [10,11].

In teleost, the first type I IFN receptor was identified in the pufferfish (*Tetraodontidae*) cytokine receptor family B (CRFB) gene, which shares commonality with the cluster of IFN receptor genes found in mammals [12,13]. Different types of CRFB genes have been characterized in teleost fishes, such as CRFB1 and CRFB5 in zebrafish (*Danio rerio*), Atlantic salmon (*Salmo salar*), and grass carp (*Ctenopharyngodon idella*), CRFB2 and CRFB5 in tilapia (*Oreochromis mossambicus*) [14,15,16], and CRFB13 and CRFB17 in Arapaima (*Arapaima giga*s) [17]. Protein structure analysis showed that two of the fibronectin type-3 (FN3) domains were conserved in fish CRFB5, whereas four FN3 domains were present in human IFNAR1 [18]. It has been proven that CRFB5 is a homolog of IFNAR1, while CRFB1 and CRFB2 are homologs of IFNAR2, as proven by sequence analysis and correlation experiments [19]. Some studies have found that virus infection or poly I:C stimulation could induce CRFB5 expression in some teleost fishes, including orange-spotted grouper (*Epinephelus coioides*), Asian Arowana (*Scleropages formosus*)*,* Nile tilapia (*Oreochromis niloticus*), and grass carp [14,15,17,18]. For example, poly I:C stimulation caused the up-regulation of CRFB5 in the liver of tilapia and Asian arowana [14,20], as well as in grass carp kidney cells (CIK cell) [15], which demonstrated that CRFB5 was involved in antiviral immune responses in fish [20]. However, whether fish CRFB5 could be induced by pathogen infection and involved in host antibacterial immunity remains unclear. Hence, in vivo and in vitro gene expression analysis of CRFB5 upon various PAMPs or pathogen infections is needed to reveal its functional regulation in teleost fish antiviral and antibacterial immune responses. So far, the majority of the immune function research on fish CRFB proteins has focused on how to recognize and interact with their corresponding IFNs. It has been documented that complexes of CRFB1 and CRFB5 binding with IFNd and IFNh, or CRFB2 and CRFB5 binding with IFNc, occur in tilapia [14]. In Asian arowana, the receptor complexes of CRFB1/CRFB5b, CRFB2/CRFB5a, and CRFB2/CRFB5b were found to interact with IFNa1 or IFNa2, IFNb, and IFNc, respectively [20]. Based on bioinformatics analysis, in grass carp, group I IFN binds to dimers of CRFB1 and CRFB5, while group II IFN binds to dimers of CRFB2 and CRFB5 [21]. Recent research has revealed that group I IFN IFNa from grass carp was found to bind to CRFB1, CRFB2, and CRFB5 [22]. However, whether fish CRFB5 is involved in the activation of known antiviral immune-related type I IFN pathway, or inflammatory signaling pathways, including NF-κB and MAPK signaling pathways induced by viral or bacterial infections, remains unclear. Therefore, more information on the immune function of CRFB5 in the control of innate immune responses is needed in teleost fishes.

In East Asian nations, the Japanese eel (*Anguilla japonica*) is a significant aquaculture species [23]. However, the frequent occurrence of diseases caused by bacterial pathogens, parasites, and especially viruses has resulted in huge economic losses for the Japanese eel farming industry [23,24,25,26]. Therefore, it is crucial to have a thorough understanding of the immunological system of this species. A homolog of CRFB5a, *Aj*CRFB5a, was first cloned from Japanese eel, and its gene expression pattern was examined in vivo and in vitro following *A. hydrophila* infection and several PAMP stimulations. Then, using *Aj*CRFB5a overexpression or RNAi in vivo and in vitro, we examined its possible functions in regulating IRF3/IRF7-mediated type I IFN and inflammation-related NF-κB and MAPK signaling pathways. These discoveries will further be understanding of CRFB5′s role in controlling inflammatory reactions to bacterial and viral infections in teleost and provide the functional investigation of CRFB5 in vertebrates fresh impetus.

## 2. Materials and Methods

### 2.1. Fish Tissue Collection and Immune Challenge

Fuqing eel farms provided healthy Japanese eels (weighing 40–45 g), which were temporarily raised in circulating water for two weeks. Different tissues were collected under natural conditions and tissue RNA was extracted to detect tissue distribution as previously studied [27]. The procedure for in vivo and in vitro immunostimulation experiments is described in a previous report from our laboratory [27]. Tryptone soy broth (TSB) was infected with *Aeromonas hydrophila*, and the mixture was shaken at 28 °C for 24 h. The bacteria were collected and diluted in 0.01 mmol/L PBS (pH 7.4) to a concentration of 4 × 10^4^ cfu/mL. Fish immune stimulation was carried out by intraperitoneal injection of 250 μL each of LPS (Sigma-Aldrich^®^, St. Louis, MO, USA, 4 mg/mL), poly I:C (Sigma, 2 mg/mL), and *A. hydrophila* (4 × 10^4^ cfu/mL) in phosphate-buffered saline (PBS). Control fish were injected with 250 μL of PBS. For each time point, four fish were slaughtered for each of the control and experimental groups. Each group’s liver, spleen, and kidney were removed and stored for a quantitative real-time polymerase chain reaction (RT-qPCR) at 0, 6-, 12-, 24-, 48-, and 72-h following injection. The day before the treatment, Japanese eel liver cells were sown in 6-well culture plates and were at a confluence of around 85%. Then, 30 mg/mL LPS (Sigma), 50 mg/mL poly I:C (Sigma), 30 mg/mL CpG-DNA (Sangon Biotech, Shanghai, China), and 30 mg/mL PGN (Sigma) were added to the cells, with the untreated cells acting as the control. Each group had four parallel samples taken at 0, 3, 6, 12, 24, and 48 h following treatment.

### 2.2. Cloning of the Full-Length cDNA of CRFB5a and Bioinformatics Analysis

The cDNA of each tissue was synthesized using a PrimeScript RT reagent Kit with gDNA Eraser (Takara, Beijing, China), as described previously [27]. The one-step cloning technique was used to create the plasmid (In-Fusion Snap Assembly Master Mix, Takara, Beijing, China). The endonucleases Xbal and BamHI (Thermo Fisher Scientific, Waltham, MA, USA) were used to doubly digest the pcDNA3.1 plasmid (Invitrogen, USA), which was then recombined with *Aj*CRFB5a to create pcDNA3.1-*Aj*CRFB5a. HindIII and BamHI (Thermo Fisher Scientific, Waltham, MA, USA) endonucleases were used to doubly digest the EGFP-N1 plasmid (Youbio, Hunan, China). The result was EGFP-*Aj*CRFB5a, which was then recombined with *Aj*CRFB5a. To extract the whole cDNA sequence of *Aj*CRFB5a, special primers (Table 1) were created. Protein 3D structures were predicted using the phyre2 online website and protein structure models were analyzed using Pymol software (Version 2.5.4, https://pymol.org, accessed on 13 January 2023). Using the MEGA 6 program and the Neighbor-joining method with a bootstrapping of 1000 iterations, a phylogenetic tree was created, and the phylogenetic tree was landscaped using the iTOL online website (https://itol.embl.de/itol.cgi, accessed on 29 January 2023). The BLAST tool was used to analyze the similarity of the sequences (http://blast.ncbi.nlm.nih.gov/Blast.cgi, accessed on 10 January 2023). Utilizing the ExPASy Molecular Biology server (http://www.us.expasy.org/tools/, accessed on 13 January 2023), the deduced amino acid sequence was examined. The CLUSTALW tool was used to align several sequences (http://www.ebi.ac.uk/clustaw/, accessed on 13 January 2023).

### 2.3. Subcellular Localization Analysis

Subcellular localization of CRFB5a in Japanese eel liver cells using electrotransfection as our previous study described [27]. HEK293 cells were cultured in DMEM medium (Gibco^TM^, Thermo Fisher Scientific, Waltham, MA, USA) containing 10% fetal bovine serum (FBS, Gibco^TM^, Thermo Fisher Scientific, Waltham, MA, USA) and antibiotics (100 IU/mL penicillin and 100 mg/mL erythromycin, Gibco) at 37 °C with a CO_2_ concentration of 5%. Japanese eel liver cells and FHM cells were cultured in DMEM/F12 medium (Gibco) containing 15% fetal bovine serum (FBS, Gibco) and antibiotics (100 IU/mL penicillin and 100 mg/mL erythromycin, Gibco) at 27 °C without CO_2_. Cells in logarithmic growth were first selected, and when cell coverage reached 70–80%, the medium was changed to a complete medium without penicillin and erythromycin one day before electrotransfection. The next day, cells were collected and suspended in 25 μL Opti MEM I (1 × 10^6^/tube) to bring the resistance to 200 ohms. Next, 3 μg of EGFP-*Aj*CRFB5a or EGFP-N1 empty vector was mixed thoroughly with cells and electroporated at 150 v/10 ms using a 2 mm electrode cup in an electroporator (BEX CUY21 EDIT II, Japan). Cells were examined under a confocal microscope and photographed (Leica TCS SP8, Leica Microsystems GmbH, Wetzlar, Germany).

### 2.4. Expression Analysis of CRFB5a by RT-qPCR

On a Roche Light Cycler 480 device (Roche, Sussex, UK), RT-qPCR was carried out using the following reaction protocol: an initial incubation at 95 °C for 1 min, followed by 40 cycles of 95 °C for 15 s and 60 °C for 1 min. For further information, please refer to our prior investigations [27]. The relative mRNA expression level of AjCRFB5a was calculated using the comparative CT method (2^−ΔΔCT^ method).

### 2.5. Luciferase Activity Assay

HEK293 cells (Human Embryonic Kidney 293 cell line) and FHM cells (Fathead Minnow muscle cell line) were used for this experiment and cells were collected 24 h after transfection for assay, as our previous study described [27]. Utilizing Lipofectamine 3000 Reagent (Invitrogen, Carlsbad, CA, USA), HEK 293 cells were transfected with 20 ng of the pRL-TK reference plasmid, 80 ng of the human IFN-luc luciferase reporter plasmid, and 300 ng of the pcDNA-CRFB5a. These cells were seeded overnight in 48-well plates at a density of 1 × 10^5^ cells per well.

### 2.6. Western Blotting

Japanese eel liver cells were used, and the proteins were extracted 24 h after transfection. The overexpressed cells were washed twice with PBS, then lysed by adding Western and IP cell lysates (RIPA, Beyotime, Shanghai, China) for 15 min on ice, and centrifuged at 12,000× *g* for 5 min, and the supernatant containing the proteins was retained. Then, 2× SDS Protein Sampling Buffer was added to the supernatant, boiled for 15 min, and stored at −80 °C for Western blot analysis. His Tag Mouse Monoclonal Antibody (Product No. AF5060, Beyotime, Shanghai, China) was used as the primary antibody and HRP-labeled Goat Anti-Mouse IgG (Product No. A0216, Beyotime, Shanghai, China) was used as the secondary antibody.

### 2.7. Overexpression of CRFB5a

To reveal the effects of CRFB5a on the expression of immune-related genes, including IFN1, IFN2, IFN3, IFN4, IRF3, IRF5, IRF7, and LEAP2, Japanese eel liver cells were seeded into 6-well plates at a density of 1 × 10^6^ cells per well for 24 h. Subsequently, pcDNA3.1-CRFB5a and pcDNA3.1 empty vectors (control) were transfected with lipo3000. Twenty-four hours later, cells were harvested to extract total RNA for preparing cDNA templates, and then the expression of the above immune-related genes was detected using RT-qPCR by specific primers listed in Table 1.

### 2.8. RNA Interference of CRFB5a In Vivo and In Vitro

Using RNA interference (RNAi), CRFB5a was silenced. Based on the ORF sequence of CRFB5a, the gene-specific primers with a T7 promoter sequence (Table 1) were designed to amplify a 341 bp fragment using the online program (https://www.flyrnai.org/cgi-bin/RNAi_find_primers.pl, accessed on 5 August 2022). The enhanced green fluorescent protein (EGFP) gene was used as an exogenous control dsRNA (dsEGFP). The detail of dsRNA synthesis referenced our previous studies, as described [28]. For a short Twelve Japanese eels weighing 20 to 25 g were separated into two groups and given intraperitoneal injections of CRFB5a dsRNA (40 g/individual) and EGFP dsRNA (40 g/individual) for 24 h, respectively, in an RNA interference experiment in vivo. Then, the tissues of the liver, spleen, kidney, and intestine were collected for RNA extraction. For an in vitro short RNA interference experiment, Japanese eel liver cells were cultured as described in our previous study [27]. The expression levels of CRFB5a, IFN1, IFN3, IFN4, IRF3, IRF5, IRF7, and LEAP2 genes in Japanese eel were detected using RT-qPCR (Table 1).

### 2.9. Statistical Analysis

SPSS 15.0 system software was used to statistically evaluate experimental data. To identify the significant difference between the two groups, the Student’s *t*-test was performed. By using a one-way analysis of variance (ANOVA) in accordance with Duncan’s multiple ranges, the significant differences between the various groups were assessed. *p* values of less than 0.05 were considered statistically significant.

## 3. Results

### 3.1. Sequence Analysis of CRFB5a from Japanese Eel

**Figure 1:**
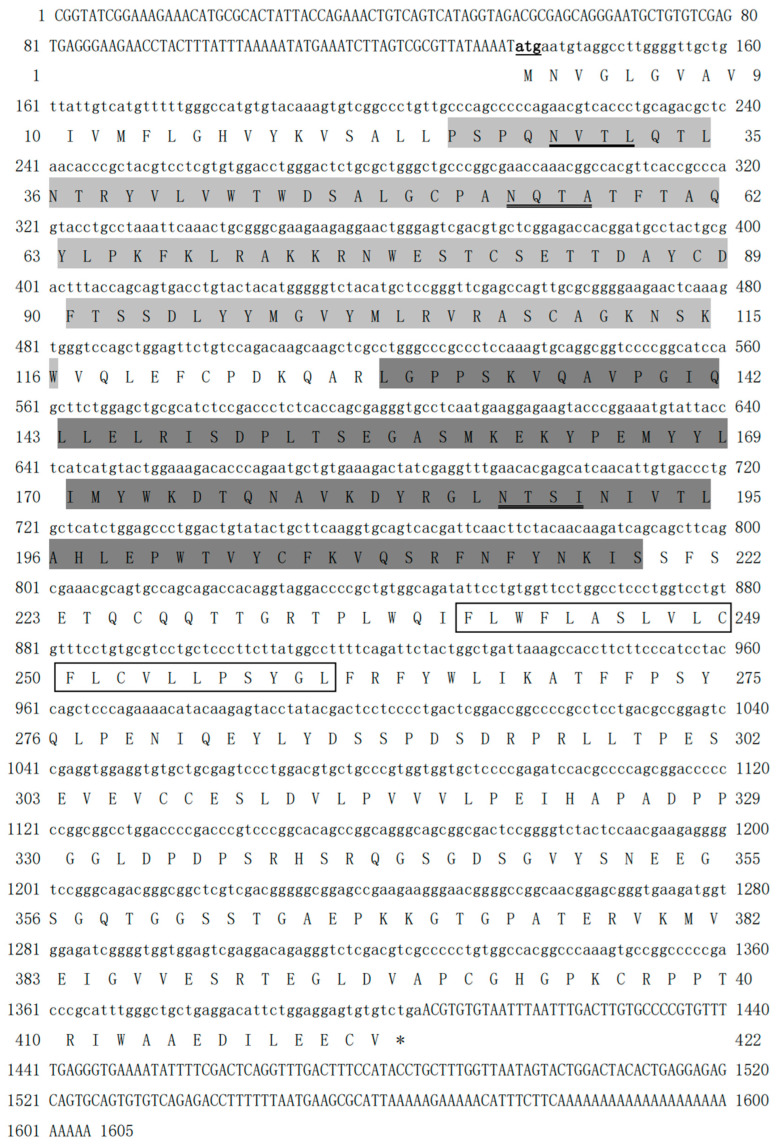
Japanese eel *Aj*CRFB5a gene cDNA and its deduced amino acid sequence. The nucleotide and amino acid sequences were numbered on the left. Light gray and dark gray represent fibronectin-3 domains, and the box represents the transmembrane region.

**Figure 2:**
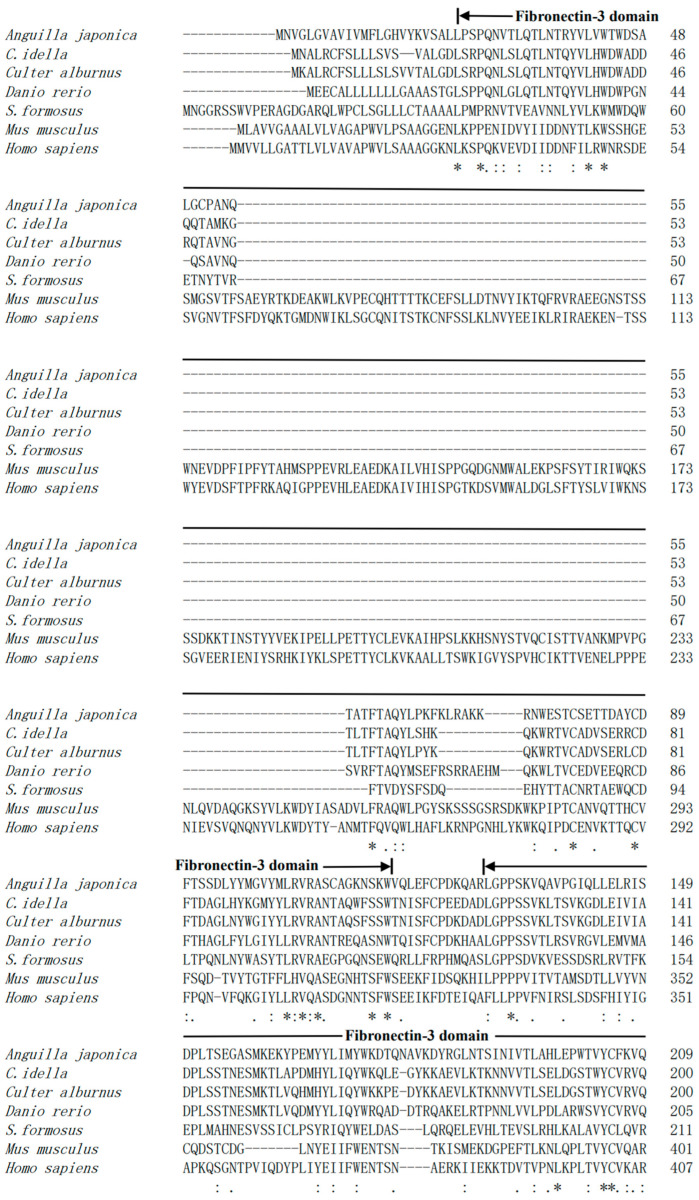

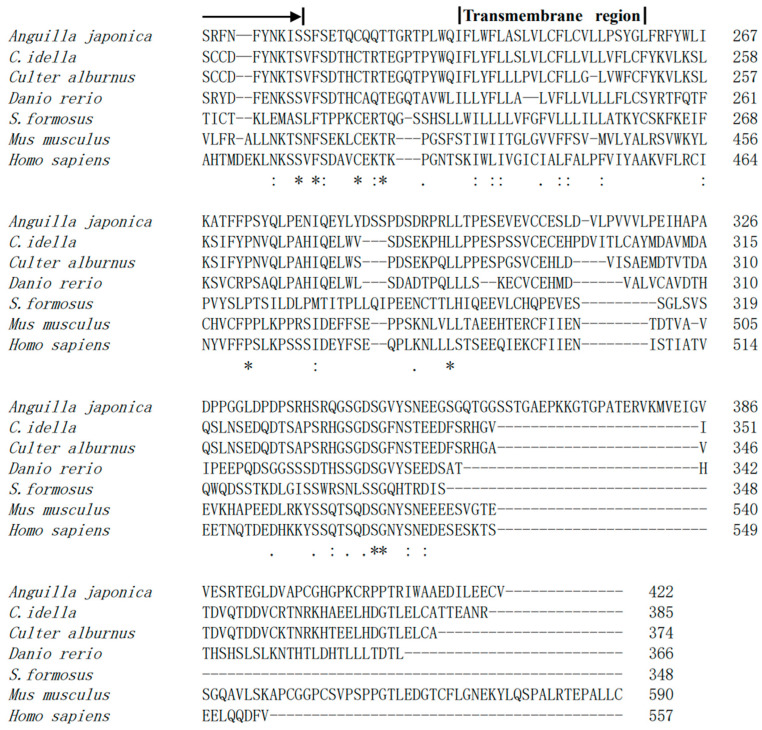
Multiple comparisons of IFNAR1 amino acid sequences of Japanese eel *Aj*CRFB5a of other species. Structural domains are indicated by arrows, identical amino acid residues are denoted by “*”; similar amino acid residues are denoted by “: or .” for similar amino acid residues.

**Figure 3:**
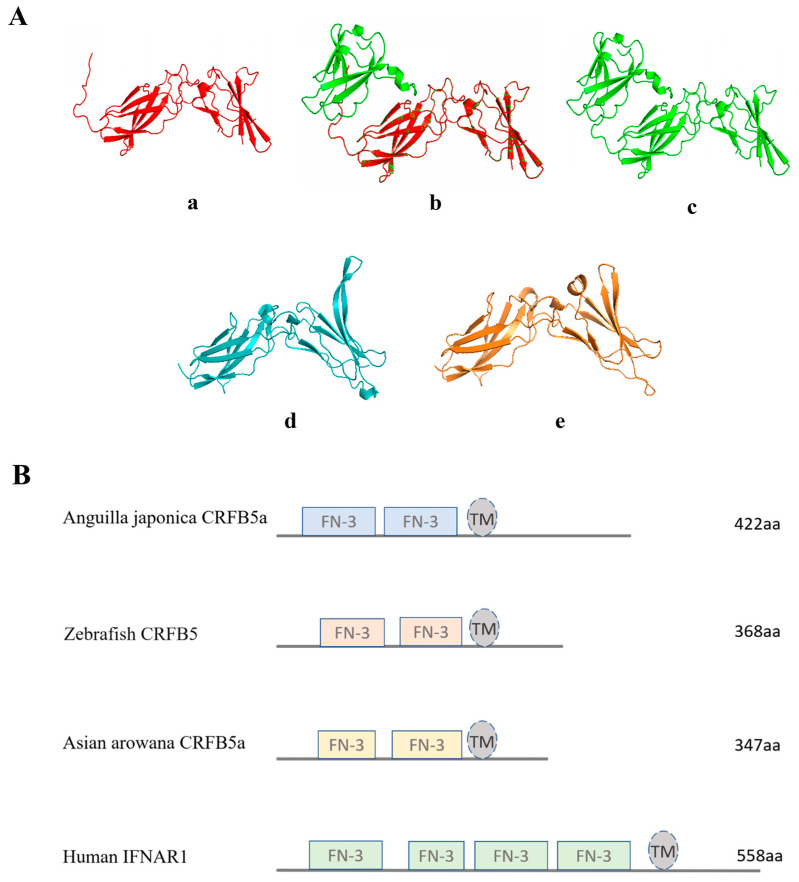
(**A**) Three-dimensional ribbon structure of IFN receptor (**a**) spatial structure simulation of Japanese eel *Aj*CRFB5a (**b**) spatial structure simulation of IFN receptor superposition, (**c**) spatial structure simulation of human IFNAR1, (**d**) spatial structure simulation of zebrafish CRFB5, (**e**) spatial structure simulation of Asian arowana CRFB5a. (**B**) The predicted conserved functional domains of *Aj*CRFB5a protein. FN-3 denotes the fibronectin type 3 structural domain, with TM as the transmembrane structural domain. The accession numbers are zebrafish (ABJ97310.1), Asian arowana (MW286829.1), and human (NP_000620.2).

**Figure 4:**
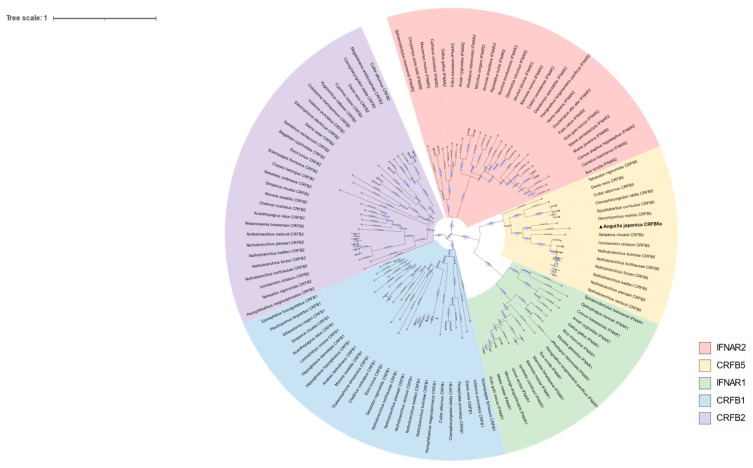
*Aj*CRFB5a’s phylogenetic tree with other CRFB5a proteins from different animals. The phylogram was built using the Neighbor-joining (NJ) method using the MEGA11 software (Version 11.0.13).

The cDNA of CRFB5a (GenBank registration number: MF537666) is 1605 bp and contains 135 bp of 5′ untranslated regions (UTR), 201 bp of 3′-UTR, and 1269 bp of ORF, which encoding 422 amino acids (aa) containing two fibronectin-3 domains (25–116 aa and 129–219 aa) and a transmembrane region (238–260 aa) (Figure 1). The fibronectin-3 domain and transmembrane region of *Aj*CRFB5a were found to be preserved when it was compared to CRFB5 from zebrafish, Asian arowana, grass carp, topmouth culter, or IFNAR1 from mice and humans. (Figure 2). The three-dimensional structure of *Aj*CRFB5a was similar to that of zebrafish and Asian arowana (Figure 3). Additionally, together with zebrafish CRFB5, *Aj*CRFB5a, and other teleost CRFB5 clustered to one branch, whereas IFNAR1 and IFNAR2 from the mammals, birds, and reptiles or CRFB1 and CRFB2 from other bony fish formed another branch (Figure 4).

### 3.2. Subcellular Localization of AjCRFB5a

**Figure 5:**
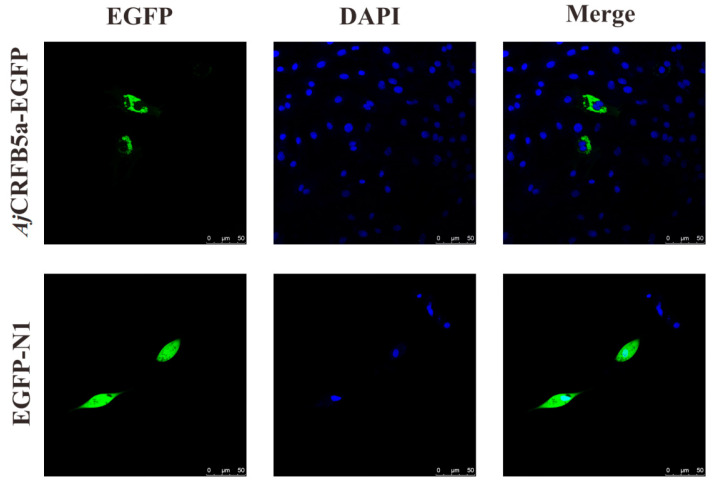
Subcellular localization of *Aj*CRFB5a. EGFP-*Aj*CRFB5a and EGFP-N1 empty vectors were electrotransfected into Japanese eel liver cells. The cells were stained with DAPI after 24 h of incubation and then examined and photographed under a confocal microscope.

Fluorescence microscopy results showed that EGFP-*Aj*CRFB5a proteins were distributed on the membranes with some of them in a condensed form in Japanese eel liver cells (Figure 5).

### 3.3. Tissue Expression of AjCRFB5a

**Figure 6:**
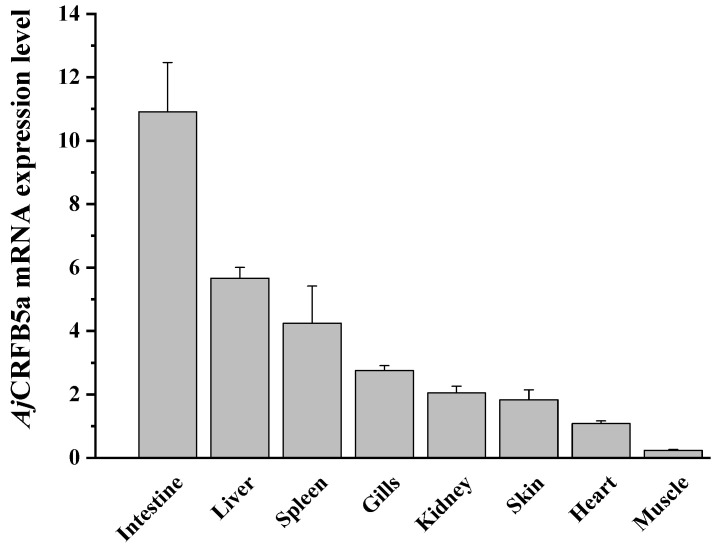
Relative expression levels of *Aj*CRFB5a gene in various tissues of healthy Japanese eel.

The outcomes demonstrated that all identified organs and tissues expressed CRFB5a, with a higher expression level in the intestine and liver, followed by the spleen, gill, and kidney, and lower expression levels in the skin, heart, and muscle (Figure 6).

### 3.4. Gene Expression of AjCRFB5a In Vivo upon Stimulation

**Figure 7:**
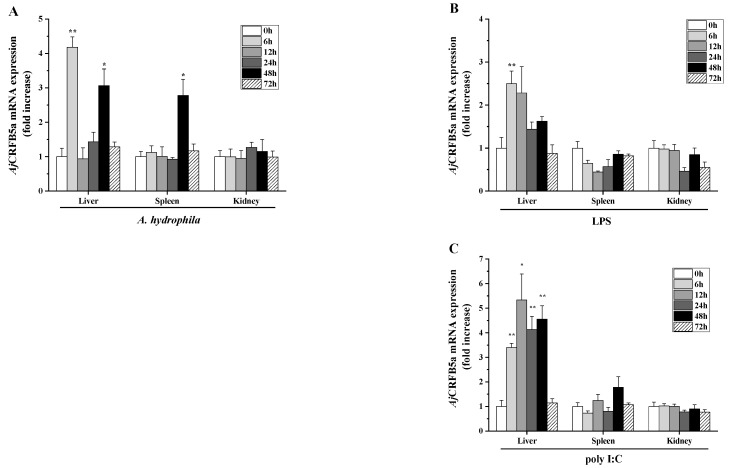
*Aj*CRFB5a gene expression in vivo in response to immunological challenge. *A. hydrophila* (**A**), LPS (**B**), or poly I:C (**C**) were intraperitoneally injected into Japanese eels, respectively. RT-qPCR was used to calculate *Aj*CRFB5a expression levels, and the fold increase was normalized to PBS control (n = 4). Statistical differences were marked with asterisks (*, *p* < 0.05; **, *p* < 0.01).

RT-qPCR analysis was used to identify the amounts of *Aj*CRFB5a gene expression in the liver, spleen, and kidney of Japanese eels stimulated with LPS, poly I:C, and *A. hydrophila*. *Aj*CRFB5a was increased following *A. hydrophila* infection, with 4.2 and 3.1 fold at 6 h and 48 h in the liver, and 2.8 fold at 48 h in the spleen (Figure 7A). Under LPS stimulation, *Aj*CRFB5a was up-regulated 2.5 fold at 6 h in the liver, but no up-regulation of *Aj*CRFB5a was detected in the kidney and spleen (Figure 7B). *Aj*CRFB5a was also induced by poly I:C, with 3.4-, 5.3-, 4.1-, and 4.6 fold at 6 h, 12 h, 24 h, and 48 h in the liver, but no up-regulation of *Aj*CRFB5a was detected in the kidney and spleen (Figure 7C).

### 3.5. Gene Expression of AjCRFB5a In Vitro after Stimulation

**Figure 8:**
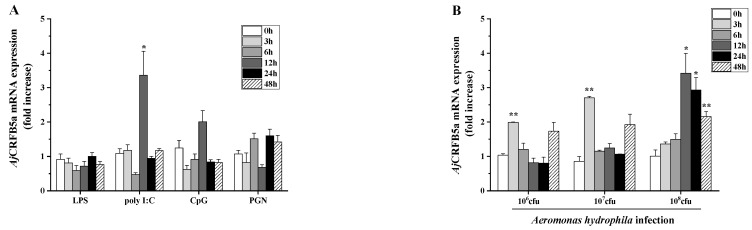
The levels of *Aj*CRFB5a gene expression in Japanese eel liver cells after PAMP stimulation or after infection with *A. hydrophila*. Japanese eel liver cells were stimulated using LPS, poly I:C, CpG-DNA, PGN (**A**), and three different doses of *A. hydrophila* or PBS as a control (**B**). *Aj*CRFB5a expression levels were determined using RT-qPCR, and the fold increase was standardized to PBS control (n = 4). Statistical differences were marked with asterisks (*, *p* < 0.05; **, *p* < 0.01).

The expression of *Aj*CRFB5a was up-regulated with 3.4-fold at 12 h upon poly I:C challenge, LPS, CpG, and PGN failed to induce CRFB5a gene (Figure 8A), or 2.0 and 2.7 fold at 3 h by the infection of 1 × 10^6^ cfu/mL, 1 × 10^7^ cfu/mL of *A. hydrophila*, and when the concentration reached 1 × 10^8^ cfu/mL, there was a 3.4-, 2.8-, and 2.2-fold increase at 12 h, 24 h, and 48 h, respectively (Figure 8B).

### 3.6. Dual Luciferase Activity of the AjCRFB5a Gene

**Figure 9:**
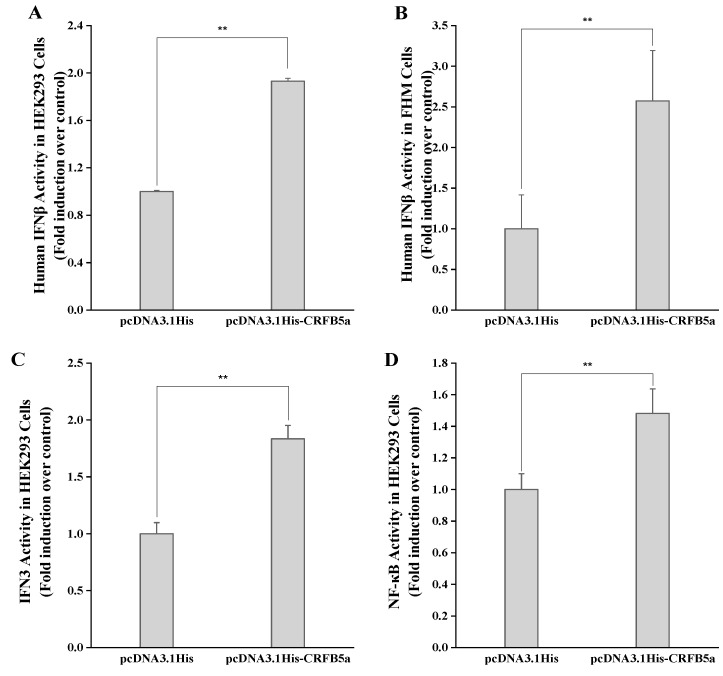
Human IFNβ response is activated by ectopic expression of *Aj*CRFB5a in HEK293 cells (**A**) and FHM cells (**B**); *Aj*IFN3 (**C**) and NF-κB (**D**) response are activated by ectopic expression of *Aj*CRFB5a in HEK293 cells. Cells cultured in 48-well plates with 1 × 10^6^ cells per well were transfected with 20 ng of pRL-TK reference plasmid, and 80 ng of luciferase reporter plasmid, plus 300 ng of pcDNA3.1His-*Aj*CRFB5a, or empty pcDNA3.1His as a control. At 24 h post-transfection, cells were harvested to assay for luciferase activity. Statistical differences in expression levels between each sample and the pcDNA3.1His control are indicated by an asterisk (**, *p* < 0.01).

To evaluate the effect of *Aj*CRFB5a on the type I IFN and NF-κB signaling pathways, we co-transfected pcDNA3.1His-*Aj*CRFB5a with human IFNβ, NF-κB luciferase reporter plasmids, and *Aj*IFN3 promoter into HEK293 cells and FHM cells. The results showed that the activity of pcDNA3.1His-*Aj*CRFB5a luciferase was significantly increased compared with pcDNA3.1His (Figure 9).

### 3.7. Induction of Immune-Related Genes under Overexpression of AjCRFB5a

**Figure 10:**
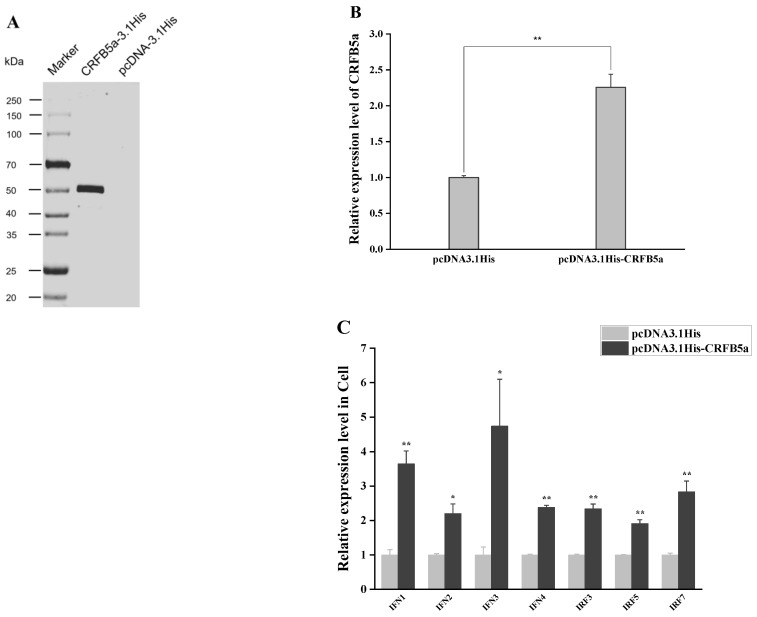
Expression pattern of immune-related genes after overexpression of CRFB5a. Japanese eel liver cells either received pcDNA3.1His-CRFB5a or pcDNA3.1His empty vector (control) during transfection. Western blot analysis using Anti-His antibody was performed to confirm the expression of pcDNA3.1His-*Aj*CRFB5a and pcDNA3.1His fusion proteins (**A**). The expression levels of CRFB5a were detected by RT-qPCR (**B**). mRNA expression levels of IFN1, IFN2, IFN3, IFN4, IRF3, IRF5, IRF7 were detected by RT-qPCR (**C**). Statistical differences in expression levels between pcDNA3.1His-CRFB5a group and pcDNA3.1His empty vector control group are indicated by asterisks (*, *p* < 0.05; **, *p* < 0.01).

The molecular weight of *Aj*CRFB5a was calculated to be 47.24 kDa by the Novopro online tool, and Western blot analysis using Anti-His antibody showed that the molecular weight of the pcDNA3.1His-*Aj*CRFB5a fusion protein was close to 50 kDa (Figure 10A). The expression profiles of *Aj*CRFB5a and immune-related genes under overexpression of *Aj*CRFB5a in Japanese eel liver cells were assayed using RT-qPCR. It was found that CRFB5a was successfully overexpressed in Japanese eel liver cells (Figure 10B). *Aj*CRFB5a overexpression significantly enhanced the expression of immune-related genes in Japanese eel liver cells, including IFN1, IFN2, IFN3, IFN4, IRF3, IRF5, and IRF7 of type I IFN signaling pathway (Figure 10C).

### 3.8. The effect of AjCRFB5a Gene Silencing on the Immune-Related Genes from Type I IFN Signaling Pathways

**Figure 11:**
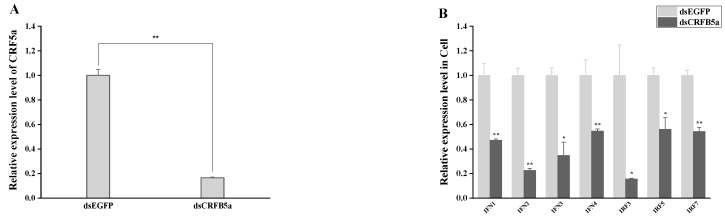
Immune-related gene expression patterns following in vitro *Aj*CRFB5a gene silencing. By using RT-qPCR to measure *Aj*CRFB5a’s mRNA expression levels in Japanese eel liver cells, the knockdown of the CRFB5a gene (**A**) was verified. The mRNA expression levels of IFN1, IFN2, IFN3, IFN4, IRF3, IRF5, IRF7 in the cells were detected by RT-qPCR (**B**). Statistical differences in expression levels between dsCRFB5a group and dsEGFP control are indicated by asterisks (*, *p* < 0.05; **, *p* < 0.01).

**Figure 12:**
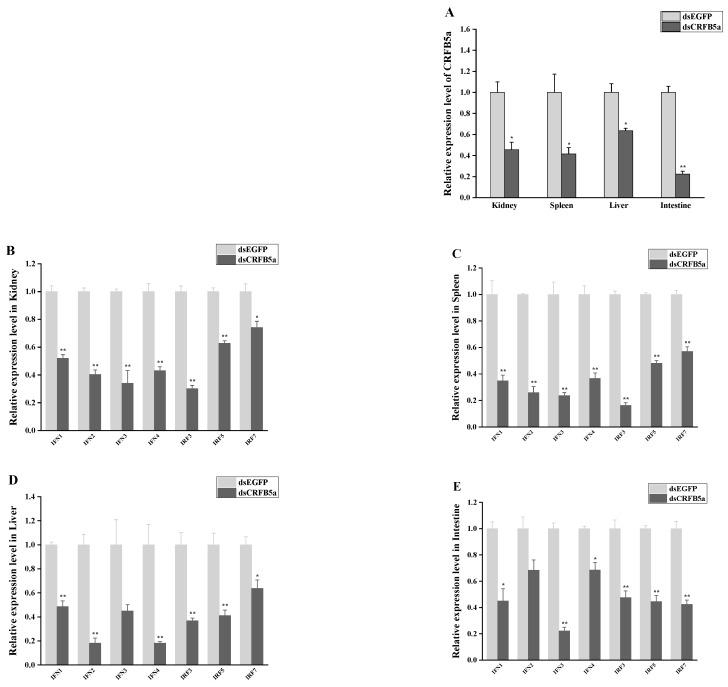
Immune-related gene expression patterns following in vivo *Aj*CRFB5a gene silencing. To verify the knockdown of the *Aj*CRFB5a gene, the levels of mRNA expression of *Aj*CRFB5a in the kidney, spleen, liver, and intestine were found using RT-qPCR (**A**). RT-qPCR was used to identify the mRNA expression levels of IFN1, IFN2, IFN3, IFN4, IRF3, IRF5, IRF7 in the kidney (**B**), spleen (**C**), liver (**D**), and intestine (**E**). Statistical differences in expression levels between dsCRFB5a group and dsEGFP control group are indicated by asterisks (*, *p* < 0.05; **, *p* < 0.01).

RT-qPCR analysis was used to assess the expression patterns of *Aj*CRFB5a as well as immune-related genes in the liver, spleen, kidney, intestine, gills, and Japanese eel liver cells. The RT-qPCR results showed that the expression of *Aj*CRFB5a in the liver cells of Japanese eel was also significantly inhibited (Figure 11A). Meanwhile, the expression of *Aj*CRFB5a was significantly inhibited in the liver, spleen, kidney, and intestine (Figure 12A). Knocking down *Aj*CRFB5a caused the expression of IFN1, IFN2, IFN3, IFN4, IRF3, IRF5, and IRF7 decreased to varying degrees in the kidney (Figure 12B), spleen (Figure 12C), liver (Figure 12D), and intestine (Figure 12E). All those genes were significantly down-regulated in Japanese eel liver cells following the inhibition of *Aj*CRFB5a (Figure 11B).

### 3.9. The Effect of AjCRFB5a on the Antimicrobial Peptide LEAP2

**Figure 13:**
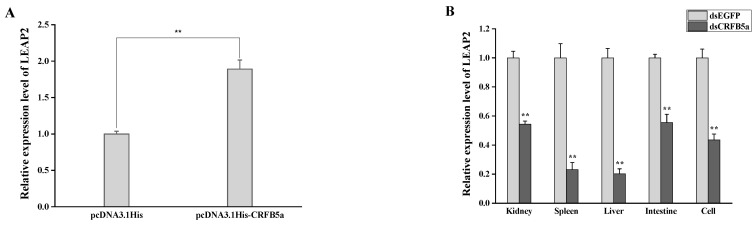
Expression patterns of the LEAP2 gene following gene overexpression (**A**) and gene silencing (**B**). Statistical differences in expression levels between experimental and control group are indicated by asterisks (**, *p* < 0.01).

The expression profiles of the key antimicrobial peptide gene LEAP2 under *Aj*CRFB5a overexpression and gene silencing were examined using RT-qPCR. The results revealed that the expression of the LEAP2 gene was significantly up-regulated after *Aj*CRFB5a was overexpressed in Japanese eel liver cells (Figure 13A). In both in vitro and in vitro experiments, LEAP2 gene expression was significantly down-regulated after *Aj*CRFB5a was knocked down in the kidney, spleen, liver, intestine, and Japanese eel liver cells (Figure 13B).

## 4. Discussion

In mammals, through binding with its receptors and triggering the JAK/STAT signaling cascade, type I IFN plays a key role in host antiviral immunity, ultimately leading to the production of Mx proteins and other antiviral proteins to prevent virus infection [6]. The immune function research of Type I IFN receptors in teleost fishes is now in the starting stage and is mainly involved in the recognition and interaction between CRFB and IFNs. However, whether fish CRFB is involved in the activation of known antiviral immune-related type I IFN pathways, or inflammatory signaling pathways including NF-κB and MAKP signaling pathways induced by viral or bacterial infections, remains largely unknown. In this study, we cloned a Japanese eel CRFB5a homolog, identified immune challenge-induced changes in its expression patterns in vivo and in vitro, and assessed the function of Japanese eel CRFB5a in regulating the activation of signaling pathways such as type I IFN, NF-κB, and MAPK signaling pathways by overexpressing or inhibiting *Aj*CRFB5a using RNAi in vivo or in vitro. The current research offers a fresh understanding of CRFB5a’s multiple functions in vertebrates’ innate immune response.

According to multiple alignments and NCBI CDD analysis, a transmembrane region and two typical FN3 domains were found in CRFB5a showing a similar three-dimensional structure to zebrafish CRFB5, Asian arowana CRFB5a, and human IFNAR1 [18,20]. Together with the finding that overexpression of *Aj*CRFB5a potently induced human IFN-β promoter activity in HEK293 cells, *Aj*CRFB5a might possess the binding affinity with its corresponding IFNs to trigger immune-related signaling pathways similar to those mammalian IFNAR1. Unlike uniformly distributed EGFP in the entire cells, *Aj*CRFB5a is mainly present in the membranes of Japanese eel liver cells, with no distribution in the nucleus, which is consistent with the findings in orange-spotted grouper [18]. The constitutive expression pattern of *Aj*CRFB5a in Japanese eel was in line with the reports from Asian arowana, orange-spotted grouper, and Dabry’s sturgeon (*Acipenser dabryanus*) [18,20,29]. However, CRFB5 was found to be highly expressed in the liver of Japanese eel and Asian arowana, and lowly in the liver of orange-spotted grouper, which indicated that CRFB5 plays different roles in immune-related tissues due to the species difference [18,20].

The synthetic dsRNA poly I:C, recognized by TLR3, RIG-I, or MDA5 in the cytoplasm of all somatic cells [30], has been widely applied to mimic RNA viral infection to initiate the type I IFN signal transduction pathway [31]. Here, we discovered that poly I:C substantially increased the expression of *Aj*CRFB5a in the liver, which was congruent with findings from Asian arowana [20]. The enhanced *Aj*CRFB5a expression was also found in Japanese eel liver cells following stimulation with poly I: C, which is similar to the findings in grass carp kidney cells [32] and Dabry’s sturgeon primary spleen leukocytes [29]. Thus, these findings collectively suggest that *Aj*CRFB5a might be involved in controlling host antiviral immune responses. In mammals, IFNAR1 and IFNAR2 were combined with IFN to activate the JAK/STAT signaling pathway, resulting in the expression of Mx proteins and other antiviral proteins [8,9]. Although the effect of type I IFN receptors on the activation of the immune-related signaling pathway remains unclear, recent studies documented that knocking down IFNAR1 in orange-spotted grouper caused a decreased number of nervous necrosis in grouper fin cells [18], and the complex of CRFB2 and CRFB5a in Asian arowana was found to combine with IFNb to enhance the gene expression of Mx and Vipein in EPC cells [20]. In order to answer the question of which mechanism is available for the elevated *Aj*CRFB5a expression contributing to antiviral responses, we determined its potential roles in the regulation of IRF3/IRF7-mediated type I IFN by overexpression or RNAi of *Aj*CRFB5a in vivo and in vitro. Our results showed that *Aj*CRFB5a overexpression significantly enhanced the expression of IFN1, IFN2, IFN3, and IFN4, as well as transcriptional factors including IRF3, IR5, and IRF7. The generation of type I interferon (IFN) in mammals is induced by the phosphorylation of IRF3 and/or IRF7, which results in their dimerization and nuclear translocation [33]. Huang et al. [34] cloned four type I IFN (IFN1, 2, 3, 4) from Japanese eel and found that overexpression of IFN1-4 induced the luciferase activity of the Mx gene reporter. Hence, *Aj*CRFB5a positively regulates host antiviral immune responses through the activation of IRF3 and IRF7-mediated type I IFN signaling pathways, resulting in the expression of Mx proteins or other antiviral proteins. Corresponding to the results of *Aj*CRFB5a overexpression in vitro, knocking down the *Aj*CRFB5a gene in the liver, spleen, and kidney of Japanese eel caused a significant decrease in expression of IFN1, IFN2, and IFN3, IFN4, IRF3, and IRF5, and IRF7 in all those tissues, which provide first-time evidence in vivo for fish CRFB involvement in the regulation of the type I IFN signaling pathway. Thus, the findings of overexpressing *Aj*CRFB5a in vitro and knocking it down in vivo demonstrated that *Aj*CRFB5a positively regulated the IRF3 or IRF7 depending on the type I IFN signaling pathway and played an important role in the antiviral immune response.

LPS is the major active component of endotoxins and performs important pathophysiological functions by activating the NF-κB signaling pathway [35]. LPS significantly increased *Aj*CRFB5a expression in the liver of Japanese eels, which is consistent with the results from orange-spotted grouper [18] and suggested that *Aj*CRFB5a might take part in the host antibacterial immune response. To clarify the role of *Aj*CRFB5a in the inflammatory response against bacterial infection, we herein used a major aquatic pathogenic bacterium, *A. hydrophila*, to challenge Japanese eel in vivo and Japanese eel liver cells in vitro. *A. hydrophila* strongly induced the expression of *Aj*CRFB5a in the Japanese eel liver and spleen, as well as the Japanese eel liver cells in vitro. This is direct proof that CRFB5 is involved in the anti-bacterial immune response of the host defense in teleost fishes, despite the fact that the data on the expression pattern of fish CRFB5 induced by bacterial infection is still limited [20]. NF-κB and AP-1 are well-known as the key elements in inflammatory responses that regulate the expression of inflammatory cytokines [35,36]. To further understand the regulatory mechanism mediated by the elevated *Aj*CRFB5a expression upon host inflammatory response against pathogen invasion, the expression level of the important liver-expressed antimicrobial peptide LEAP2 was analyzed under the overexpression and RNAi of *Aj*CRFB5a in vivo or in vitro. In Japanese eel liver cells, *Aj*CRFB5a overexpression significantly up-regulated LEAP2 [37]. The results suggest that *Aj*CRFB5a is able to produce antimicrobial immunity in the host by activating LEAP2.

## 5. Conclusions

In conclusion, the CRFB5a gene was identified in Japanese eels, and poly I:C stimulation and *A. hydrophila* infection can increase the gene’s expression. *Aj*CRFB5a functions in the host immune response to bacterial infection by activating antimicrobial peptide LEAP2, and it works as a positive regulator of host antiviral immune responses by activating IRF3 and IRF7-mediated type I IFN signaling pathways.

## Figures and Tables

**Table 1:** Primers used for CRFB5a gene cloning and expression analysis.

Target Gene	Primer Sequence
Specific primers for head-to-toe PCR	
CRFB5a-ORF5′	5′-ATGAATGTAGGCCTTGGGGTTG-3′
CRFB5a-ORF3′	5′-GACACACTCCTCCAGAATGTCCTC-3′
Specific primers for 5′ RACE	
CRFB5a-5-inner	5′-GTGAAGGTGATGCTCTGATTGGC-3′
CRFB5a-5-outer	5′-CGGAGGGGAAGAAGGTGGATTTGA-3′
Specific primers for RT-qPCR	
5′real-CRFB5a	5′-CGAGGTTTGAACACGAGCATC-3′
3′real-CRFB5a	5′-TCTGCTGGCACTGCGTTTC-3′
5′real-IFN1	5′-CTTGCAGGTTGAGGAACGCAT-3′
3′real-IFN1	5′-GCATTCTTTCAGCTCCGAAGC-3′
5′real-IFN2	5′-CTCAGTGAGATGGGTGGAGA-3′
3′real-IFN2	5′-GCTCGCAGAAGAAACACATTTC-3′
5′real-IFN3	5′-TGACATCACGAGCAAATCTCAG-3′
3′real-IFN3	5′-CTCGAACACACTGCTCCAAGT-3′
5′real-IFN4	5′-GCTGGGCAGAGCGACATCATT-3′
3′real-IFN4	5′-AGAATCGTCACTCCCTGGCTT-3′
5′real-IRF3	5′-CCAGGCACACCAAGCGAGTTC-3′
3′real-IRF3	5′-ACCTCTTGAGGAATGTTGCTGTTCG-3′
5′real-IRF5	5′-CCAGGTGATTCCCGTCGTTGC-3′
3′real-IRF5	5′-CCAGGTGATTCCCGTCGTTGC-3′
5′real-IRF7	5′-AGATGCGTATGCGACCGATTGC-3′
3′real-IRF7	5′-ATCATCCTGCTGCTGGTTGTTCAG-3′
5′real-LEAP-2	5′-ACTGCCTGCGGTTTGGTGTTG-3′
3′real-LEAP-2	5′-GCTTGCTTCCCATGATCCTCCAC-3′
Specific primers for dsRNA preparation	
5′dsCRFB5a	5′-TGGGGTTGCTGTTATTGTCA-3′
3′dsCRFB5a	5′-TGGGGTTGCTGTTATTGTCA-3′
5′dsCRFB5a-T7	5′-TAATACGACTCACTATAGGGTGGGGTTGCTGTTATTGTCA-3′
3′dsCRFB5a-T7	5′-TAATACGACTCACTATAGGGTGGGGTTGCTGTTATTGTCA-3′
5′dsEGFP	5′-GGTGAACTTCAAGATCCGCC-3′
3′dsEGFP	5′-CTTGTACAGCTCGTCCATGC-3′
5′dsEGFP-T7	5′-TAATACGACTCACTATAGGGGGTGAACTTCAAGATCCGCC-3′
3′dsEGFP-T7	5′-TAATACGACTCACTATAGGGCTTGTACAGCTCGTCCATGC-3′

## Data Availability

The datasets used or analyzed during the current study are available from the corresponding author upon reasonable request.
